# Differentially co‐expressed myofibre transcripts associated with abnormal myofibre proportion in chronic obstructive pulmonary disease

**DOI:** 10.1002/jcsm.13473

**Published:** 2024-04-22

**Authors:** Joe W. Chiles, Ava C. Wilson, Rachel Tindal, Kaleen Lavin, Samuel Windham, Harry B. Rossiter, Richard Casaburi, Anna Thalacker‐Mercer, Thomas W. Buford, Rakesh Patel, J. Michael Wells, Marcas M. Bamman, Beatriz Y. Hanaoka, Mark Dransfield, Merry‐Lynn N. McDonald

**Affiliations:** ^1^ Division of Pulmonary, Allergy, and Critical Care Medicine, Department of Medicine University of Alabama at Birmingham Birmingham AL USA; ^2^ Department of Epidemiology, School of Public Health University of Alabama at Birmingham Birmingham AL USA; ^3^ School of Medicine University of Alabama at Birmingham Birmingham AL USA; ^4^ Florida Institute for Human & Machine Cognition Pensacola FL USA; ^5^ Division of Trauma and Acute Care Surgery, Department of Surgery University of Alabama at Birmingham Birmingham AL USA; ^6^ Institute of Respiratory Medicine and Exercise Physiology Lundquist Institute for Biomedical Innovation at Harbor—UCLA Medical Center Torrance CA USA; ^7^ Department of Cell, Developmental, and Integrative Biology University of Alabama at Birmingham Birmingham AL USA; ^8^ Birmingham/Atlanta Geriatric Research Education and Clinical Center Birmingham Veterans Affairs Medical Center Birmingham AL USA; ^9^ Division of Gerontology, Geriatrics, and Palliative Care, Department of Medicine University of Alabama at Birmingham Birmingham AL USA; ^10^ Department of Pathology University of Alabama at Birmingham Birmingham AL USA; ^11^ Birmingham Veterans Affairs Healthcare System Birmingham AL USA; ^12^ Department of Medicine University of Oklahoma Health Sciences Center Oklahoma City OK USA; ^13^ Department of Genetics, School of Medicine University of Alabama at Birmingham Birmingham AL USA

**Keywords:** COPD, fibre‐type shift, myofibre proportions, sex differences, skeletal muscle, transcriptomics

## Abstract

**Background:**

Skeletal muscle dysfunction is a common extrapulmonary manifestation of chronic obstructive pulmonary disease (COPD). Alterations in skeletal muscle myosin heavy chain expression, with reduced type I and increased type II myosin heavy chain expression, are associated with COPD severity when studied in largely male cohorts. The objectives of this study were (1) to define an abnormal myofibre proportion phenotype in both males and females with COPD and (2) to identify transcripts and transcriptional networks associated with abnormal myofibre proportion in COPD.

**Methods:**

Forty‐six participants with COPD were assessed for body composition, strength, endurance and pulmonary function. Skeletal muscle biopsies from the *vastus lateralis* were assayed for fibre‐type distribution and cross‐sectional area via immunofluorescence microscopy and RNA‐sequenced to generate transcriptome‐wide gene expression data. Sex‐stratified *k*‐means clustering of type I and IIx/IIax fibre proportions was used to define abnormal myofibre proportion in participants with COPD and contrasted with previously defined criteria. Single transcripts and weighted co‐expression network analysis modules were tested for correlation with the abnormal myofibre proportion phenotype.

**Results:**

Abnormal myofibre proportion was defined in males with COPD (*n* = 29) as <18% type I and/or >22% type IIx/IIax fibres and in females with COPD (*n* = 17) as <36% type I and/or >12% type IIx/IIax fibres. Half of the participants with COPD were classified as having an abnormal myofibre proportion. Participants with COPD and an abnormal myofibre proportion had lower median handgrip strength (26.1 vs. 34.0 kg, *P* = 0.022), 6‐min walk distance (300 vs. 353 m, *P* = 0.039) and forced expiratory volume in 1 s‐to‐forced vital capacity ratio (0.42 vs. 0.48, *P* = 0.041) compared with participants with COPD and normal myofibre proportions. Twenty‐nine transcripts were associated with abnormal myofibre proportions in participants with COPD, with the upregulated *NEB*, *TPM1* and *TPM2* genes having the largest fold differences. Co‐expression network analysis revealed that two transcript modules were significantly positively associated with the presence of abnormal myofibre proportions. One of these co‐expression modules contained genes classically associated with muscle atrophy, as well as transcripts associated with both type I and type II myofibres, and was enriched for genetic loci associated with bone mineral density.

**Conclusions:**

Our findings indicate that there are significant transcriptional alterations associated with abnormal myofibre proportions in participants with COPD. Transcripts canonically associated with both type I and type IIa fibres were enriched in a co‐expression network associated with abnormal myofibre proportion, suggesting altered transcriptional regulation across multiple fibre types.

## Introduction

Chronic obstructive pulmonary disease (COPD) is a leading cause of death worldwide with its impact continuing to rise.^1^ Although a diagnosis of COPD requires demonstration of impaired lung function, extrapulmonary manifestations of COPD are common and include alterations of skeletal muscle physiology.^2^ Abnormal skeletal muscle fibre proportion in COPD is one such alteration associated with worse lung function, reduced capacity for physical exertion and even mortality, but its pathophysiology remains poorly understood.^2^, ^3^, ^4^, ^5^

Generally, human skeletal muscle is composed of type I, type IIa and type IIx/IIax fibres, which can be differentiated on the basis of their expressed myosin heavy chain isoforms, as well as unique isoforms of other sarcomeric components, such as tropomyosin.^6^ There are recognized sex differences in muscle fibre‐type distribution, with females generally having more type I fibres and males having more type II fibres in health^7^ and in COPD.^8^ Ageing is typically associated with a shift towards type I fibres and away from type IIa fibres,^9^ but some studies do not find evidence of this pattern.^7^, ^10^ Skeletal muscle fibre‐type distributions have been associated with disease severity in COPD, where the term ‘fibre‐type shift’ is often used to describe an abnormal myofibre proportion predominantly due to increased type IIx/IIax fibres. Specifically, in a meta‐analysis of 22 studies by Gosker et al.,^2^ lower *vastus lateralis* type I fibre proportion and higher type IIx/IIax fibre proportion were associated with lower values of forced expiratory volume in 1 s (FEV_1_), a measure of COPD severity. The authors of this meta‐analysis defined abnormal myofibre proportion in participants with COPD as ‘a proportion of fibre type I <27% and of fibre type IIx >29%’.^2^ The populations of participants included in this meta‐analysis were overwhelmingly male,^2^ limiting generalizability to women with COPD.^2^, ^3^ Although more recent studies have included more females with COPD, the effect of sexual dimorphism in myofibre proportions on the definition of an abnormal myofibre proportion phenotype has not been specifically evaluated.^4^

Research examining differential gene expression in skeletal muscle has provided insights to the aetiology of abnormal myofibre proportions in COPD. Canonically, skeletal muscle fibres are distinguished by expression of specific myosin heavy chain (MYH) genes with type I fibres expressing *MH7*, type IIa fibres expressing *MYH2* and type IIx expressing *MYH1*.^6^ Recent research on the fibre type‐specific transcriptome has expanded the number of marker genes differentiating type I and type IIa fibres.^11^ Although this larger set of marker genes reliably differentiated 18 type I and type IIa human myofibre samples, in four samples (one type IIa and three type I), the expression fell between the two fibre types.^11^ This prior work seems to indicate that the canonical markers used to classify type I and type II myofibres may be overly simplistic and tend to leave out hybrid populations and type IIx fibres, which are not abundant in healthy individuals but are seen in higher proportions in COPD patients with abnormal myofibre proportions.

Previous research into the effects of COPD on skeletal muscle gene expression utilized Affymetrix arrays to compare participants with and without COPD.^12^, ^13^ A set of 42 genes were identified as being differentially expressed among COPD participants with low fat‐free mass index (FFMI), many of which were also correlated with type II fibre proportions.^12^ Another study identified 18 genes with large fold difference (>2‐fold) between participants with and without COPD; however, the relationship with skeletal muscle myofibre proportions was not reported.^13^ Methodological advances in next‐generation sequencing enable routine transcriptome‐wide characterization via RNA sequencing (RNA‐Seq), which offers improved specificity and a broader dynamic range than microarray.^14^ RNA‐Seq has the potential to provide insight to the underlying pathophysiology of abnormal myofibre proportion phenotype and its relationship to disease progression in participants with COPD.

In this study, we characterized sex‐stratified abnormal skeletal muscle fibre proportions in male and female participants with COPD separately, compared the discriminating power of the sex‐stratified approaches with previously proposed cut‐offs and performed transcriptomic analyses. Our results demonstrate sex‐specific criteria for abnormal myofibre proportions in participants with COPD and identify single transcripts and co‐expression networks associated with this abnormal myofibre proportion phenotype in COPD.

## Methods

### Ethical conduct of research

All research and recruitment protocols were approved by the University of Alabama at Birmingham (UAB) and Birmingham Veterans Affairs Healthcare System (BVAHS) institutional review boards. Participants provided written informed consent upon enrolment and were permitted to withdraw from the study at any time.

### Study design

Forty‐six subjects between 40 and 80 years of age with COPD were recruited as part of the COPD Cachexia: Etiology of Low Lean muscle (CCELL) study and included in these analyses. COPD was defined as a FEV_1_‐to‐forced vital capacity (FEV_1_/FVC) ratio <0.7 on post‐bronchodilator lung function testing in current or former smokers. The severity of expiratory flow limitation was characterized according to Global Initiative for Chronic Obstructive Lung Disease (GOLD) criteria.^15^ Participants completed health questionnaires, spirometry and assessment of physical function and underwent a muscle biopsy of the *vastus lateralis*. Muscle histology data from 45 healthy, sedentary, age‐matched and sex‐matched controls were leveraged from deidentified datasets provided by prior research studies.^7^ Body composition data, including FFMI, appendicular skeletal muscle index (SMI) and fat mass index (FMI), all measured as kilograms over height in metres squared, were generated on all participants using dual‐energy X‐ray absorptiometry (DEXA). Handgrip strength was assessed using a Jamar dynamometer (Patterson Companies, Saint Paul, MN). The 30‐s sit‐to‐stand test was administered by counting the number of times a participant could rise to stand from a seated position in 30 s without using their arms, which assessed functional capacity in the lower extremities.

### Muscle biopsy and histology

Skeletal muscle biopsies were taken from the *vastus lateralis* muscle using a Bergström biopsy needle under suction. After removing visible adipose, blood and connective tissue under a dissecting microscope, portions (10–30 mg) for molecular analyses (e.g., RNA‐Seq) were quickly snap frozen in liquid nitrogen. For histology, a portion of the biopsy tissue was mounted on cork in a mixture of optimal cutting temperature compound (Sakura Finetek, PA) and tragacanth gum, frozen in isopentane cooled to the temperature of liquid nitrogen and subsequently stored at −80°C. Cross‐sections of 6‐μm thickness were prepared. After appropriate fixation and blocking, sections were immunolabelled with monoclonal mouse anti‐MHCI (anti‐MHCI mouse mAb NCL‐MHCs, Novocastra Laboratories, Deer Park, IL), anti‐laminin (anti‐laminin mouse mAb VP‐L551, Novocastra Laboratories, 1:80, Deer Park, IL) and anti‐MHCIIa (anti‐MHCIIa mouse mAb, University of Iowa Hybridoma Bank, Iowa City, IA) primary antibodies, which were then conjugated with secondary fluorescent goat anti‐mouse antibodies. High‐resolution immunofluorescence images were captured and then analysed using Image‐Pro Plus 5.0 software (Media Cybernetics, Rockville, MD). The fibre‐type proportion was manually assessed based on the staining pattern. Where insufficient staining of either primary type was detectable (i.e., a negative stain for both type I and type IIa), a fibre was assigned to the IIx/IIax category. Myofibre‐type distribution was determined from an average of 965 (range 317–2213) myofibres per sample. The fibre cross‐sectional area (CSA) was measured on a minimum target of 50 randomly selected fibres of each type in each sample. In three cases where a very low fibre‐type proportion resulted in fewer than 50 myofibres available for CSA measurement, the threshold was reduced to 25 myofibres. If fewer than 25 myofibres of a given type were present in a sample, CSA data for this fibre type were considered unreliable and not used in analysis. Statistical comparisons of demographics, body composition and histology measurements between participants with COPD and age‐ and sex‐matched controls were conducted using Student's *t* tests for continuous variables and Fisher's exact tests for categorical variables.

### *k*‐means clustering

All statistical analyses were performed using R (Version 3.6.0; www.r‐project.org). *k*‐means clustering was performed using the R library ‘cluster’. COPD skeletal muscle samples were clustered into two groups based on their proportions of type I and type IIx/IIax fibres. Per cent type IIa fibres were not included in the clustering approach, as this is typically the dominant myofibre type in human *vastus lateralis* muscle. *k*‐means clustering categorized COPD participants into two groups: (1) those consistent with an abnormal myofibre proportion phenotype, characterized by a higher type IIx/IIax proportion and a lower type I proportion, compared with (2) those with a more typical muscle phenotype. We then repeated this approach in a sex‐stratified manner (i.e., in male and female COPD participants separately) to reduce the effect of fibre‐type sexual dimorphism on cluster membership. ‘Cluster’ and ‘sex‐stratified’ are used throughout this manuscript to denote the two different clustering strategies. Each participant was assigned a non‐stratified and stratified *k*‐means phenotype, either normal or abnormal. The results of these clustering methods were compared against classification into normal and abnormal groups based on the fibre‐type proportion cut‐offs proposed by Gosker et al.^2^ Demographics, functional outcomes, body composition and pulmonary function variables were compared between fibre proportion phenotypes using Mann–Whitney *U* tests for continuous variables and Fisher's exact tests for categorical variables. Linear regressions of functional outcomes against individual myofibre proportions, with and without sex as a covariate, were conducted as sensitivity analyses.

### Generation of transcriptomics data

RNA‐Seq data were generated from *vastus lateralis* muscle biopsies from participants with COPD. Briefly, ~10 mg of muscle tissue was weighed and placed into a 2‐mL Lysing Matrix D tube (MP Biomedicals, Irvine, CA, Cat. No. 6913100) along with 400 μL of RL buffer from the Norgen total RNA extraction kit (Norgen Biotek, Thorold, ON, Cat. No. 37500) plus 1% beta‐mercaptoethanol. Samples were homogenized on a MP Biomedicals FastPrep machine setting: 6.5 m/s for 45 s for three rounds. Each sample was digested using 240‐μg proteinase K (Norgen Biotek, Cat. No. 28229). RNA was extracted using the Norgen total RNA extraction kit including the DNase kit (Norgen Biotek, Cat. Nos. 37500 and 25720). RNA quality numbers (RQNs) were measured using Fragment Analyzer (Agilent, Santa Clara, CA), and RNA was quantified using a Qubit 2.0 Fluorometer (Thermo Fisher, Waltham, MA): One sample with an RQN of 1 was excluded from analysis. rRNA‐depleted RNA was used as input to the NEBNext Ultra RNA Library Prep Kit for Illumina (New England Biolabs, Ipswich, MA, Cat. No. E7530S). Libraries were barcoded using the Genomic Services Lab at HudsonAlpha's custom dual‐index barcode plate. We pooled all samples onto one lane of the S4 NovaSeq flow cell and sequenced a median of 35.5 million reads per sample.

### Transcript‐level analysis

Twenty‐nine of the 46 COPD participants with phenotype and skeletal muscle histology data also had RNA‐Seq data available and were included in the transcriptomics analysis. Raw sequencing data were cleaned and trimmed using Trim Galore! (Version 0.6.7), aligned using STAR (Version 2.7.10a),^16^ and aggregated using Picard Tools (Version 2.9.2).^17^ Counts were measured using HTseq‐count (Version 2.0.2).^18^ The data were filtered to only retain transcripts with >1 count per million in 10 or more samples. An MA plot indicated that all differentially expressed transcripts had log_2_ mean expressions >4 (*Figure* *S1*). Sequencing data were evaluated and corrected for unmeasured surrogate variables, such as batch effects, using the svaseq R package.^19^ Comparisons of count data were performed using linear regression models in the limma R package^20^ to determine whether each transcript was associated with the sex‐stratified abnormal myofibre proportion phenotype from our clustering analysis. Empirical Bayes methods were used to generate false discovery rate (FDR) *P* values based on the number of transcripts included in the analysis (*N* = 28 709). Significantly differentially expressed transcripts were queried against known gene sets using Gene Set Enrichment Analysis (GSEA) as implemented in functional mapping and annotation (FUMA) of the genome‐wide association study (GWAS) platform,^21^ using gene sets as defined by the Molecular Signatures Database (MSigDB).^22^ Although FUMA was originally designed to annotate, prioritize visualize and interpret GWAS results, its GENE2FUNC function takes in gene IDs and can be used to annotate genes in biological context.

We built custom gene sets for genes predominantly expressed in type I and type IIa skeletal muscle fibres based on prior research and included these in GSEA.^11^ We performed a series of sensitivity analyses to assess the robustness of our transcriptomic results: (1) using type I, type IIa and type IIx/IIax fibre proportions as continuous phenotypes; (2) comparing against abnormal myofibre proportion as previously defined by Gosker et al.; (3) adjusting for current smoking; and (4) performing the transcriptomic analysis within male subjects only.

### Weighted gene co‐expression network analysis

Due to the stringent *P*‐value threshold used as part of correction for multiple testing in transcriptome analysis, dimensionality‐reducing techniques can be useful in identifying patterns of co‐expressed genes. Weighted gene co‐expression network analysis (WGCNA) is one well‐studied option that uses pairwise correlations to partition the total set of genes into distinct, non‐overlapping modules, labelled by colour.^23^ Each gene in a module is assigned a level of module membership (kME) that measures the correlation of one gene's expression to the overall module's expression. The weighted expression level of each module for each sample can then be correlated with a trait of interest with less stringent *P*‐value corrections as there are fewer independent tests being conducted. The WGCNA R package was used to identify modules of genes with similar expression patterns.^23^ Because individual transcripts with variance <0.1 are unlikely to significantly contribute to co‐expression networks,^23^ these were removed, resulting in 25 770 remaining transcripts for inclusion in our WGCNA analysis. A signed correlation network was built using biweight mid‐correlation with a soft thresholding power of 7, as determined by an approximation of scale‐free topology. Transcriptomic modules generated in the overall cohort were tested for Pearson correlation with the sex‐stratified abnormal myofibre proportion phenotype from clustering analysis. Module–trait correlations were considered significant if *P* < 0.05. Networks of genes with the highest module membership in WGCNA modules were visualized using Cytoscape^24^ and evaluated for gene set enrichment using FUMA.

## Results

### Participant characteristics

Participants with COPD (*n* = 46) and controls (*n* = 45) were age‐ and sex‐matched by design (*Table* *1*). Participants with COPD tended to have appendicular SMI (7.06 ± 1.57 vs. 7.49 ± 1.16 kg/m^2^, *P* = 0.15) and FFMI (15.7 ± 2.71 vs. 16.4 ± 1.93 kg/m^2^, *P* = 0.19, *Table* *1*) compared with control participants. As transcriptomic analyses were performed on a subset of 29 participants with RNA‐sequencing (RNA‐Seq) data available, demographic and body composition metrics were compared between COPD participants included in the transcriptomics analyses and the full cohort. The COPD participants included in the transcriptomics analyses were generally similar to the full COPD cohort (*Table* *S1*).

**Table 1:** Comparison of control and COPD participants from the CCELL study

	Control (*N* = 45)	COPD (*N* = 46)	*P* value
Demographics			
Age (years)	62.6 (6.37)	63.8 (6.64)	0.39
Males, *n* (%)	28 (62%)	29 (63%)	1.00
Anthropometrics			
BMI (kg/m^2^)	25.77 (2.63)	25.32 (4.80)	0.58
SMI (kg/m^2^)	7.49 (1.16)	7.06 (1.57)	0.15
FFMI (kg/m^2^)	16.39 (1.93)	15.73 (2.71)	0.19
Pulmonary function			
FEV_1_, % pred.	—	48.33 (15.4)	—
FEV_1_/FVC	—	0.46 (0.12)	—
GOLD stages			
GOLD 2, *n* (%)	—	18 (39%)	—
GOLD 3, *n* (%)	—	25 (54%)	—
GOLD 4, *n* (%)	—	3 (7%)	—
Muscle histology			
Type I per cent	35.5 ± 10.3	30.8 ± 16.9	0.12
Type IIa per cent	49.6 ± 7.6	56.0 ± 13.3	0.006*
Type IIx/IIax per cent	14.9 ± 10.9	13.2 ± 12.0	0.47
Type I CSA (μm^2^)	4626 ± 1271	4928 ± 1840	0.36
Type IIa CSA (μm^2^)	4131 ± 1349	4627 ± 1615	0.12
Type IIx/IIax CSA (μm^2^)	3080 ± 1343	3473 ± 1541	0.28

*Note*: All data are presented as mean (standard deviation) unless otherwise noted.

Abbreviations: BMI, body mass index; CCELL, COPD Cachexia: Etiology of Low Lean muscle; COPD, chronic obstructive pulmonary disease; CSA, cross‐sectional area; FEV_1_, % pred., forced expired volume in 1 s, per cent of predicted value; FEV_1_/FVC, forced expiratory volume in 1 s‐to‐forced vital capacity ratio; FFMI, fat‐free mass index; GOLD, Global Initiative for Chronic Obstructive Lung Disease; SMI, skeletal muscle index.

* Significant difference between groups.

### Muscle fibre‐type proportions and cross‐sectional areas are sexually dimorphic

Participants with COPD had a greater percentage of type IIa fibres than controls (56.0 ± 13.3% vs. 49.6 ± 7.6%, *P* = 0.006, *Table* *1*). There were no significant differences in terms of type I fibre per cent, type IIx/IIax fibre per cent and fibre CSA of any fibre type between those with and without COPD (*Table* *1*).

**Figure 1:**
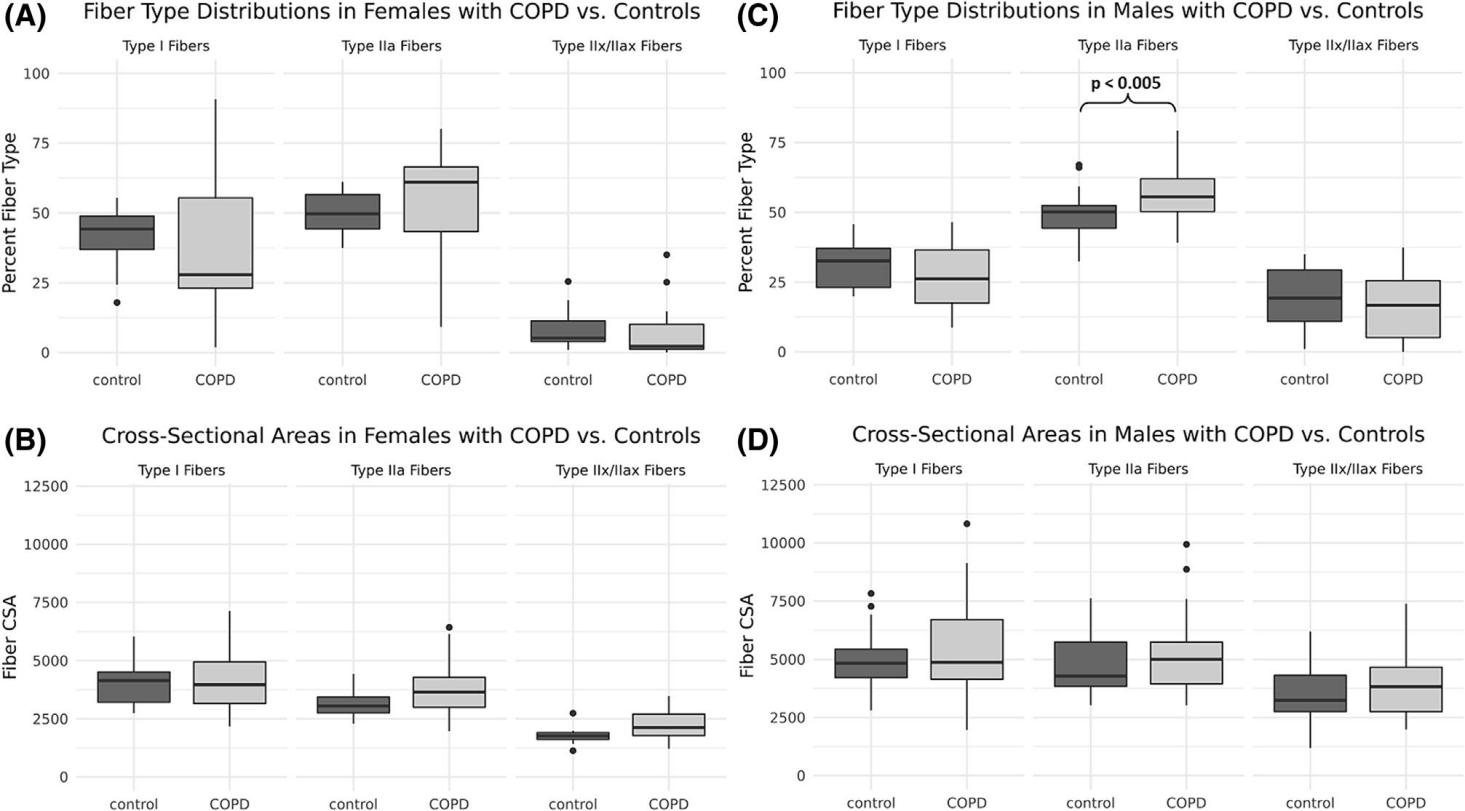
Comparisons of muscle fibre‐type proportions (A, C) and cross‐sectional areas (CSAs [μm^2^]) (B, D) between female (A, B) and male (C, D) participants on the basis of disease status. Significant differences between groups are marked with horizontal brackets and *P* values; all other comparisons were non‐significant. COPD, chronic obstructive pulmonary disease.

In comparisons between sexes, males with and without COPD had a greater percentage of type IIx/IIax and larger CSAs across all fibre types than females with and without COPD (*Figure* *1* and *Table* *S2*). Among control participants, the percentage of type I fibres was significantly less in males than females (31.6 ± 8.31% vs. 41.8 ± 10.3%, *P* = 0.001, *Table* *S2*). Among participants with COPD, a similar trend for fewer type I fibres in males versus females was not statistically significant (*Table* *S2*).

In males, participants with COPD had a greater proportion of type IIa fibres than controls (56.1 ± 8.9% vs. 49.2 ± 7.9%, *P* = 0.003, *Figure* *1* *C*). In females, fibre‐type distributions and CSAs did not differ between participants with COPD and controls (*Figure* *1* *A*,*B*). There was a trend towards greater type IIa fibre CSA in female participants with COPD in comparison with controls (3827 ± 1296 vs. 3146 ± 582 μm^2^, *P* = 0.061, *Table* *S2*).

### Abnormal myofibre proportion by sex‐stratified clustering identifies differences in function and expiratory flow limitation

**Figure 2:**
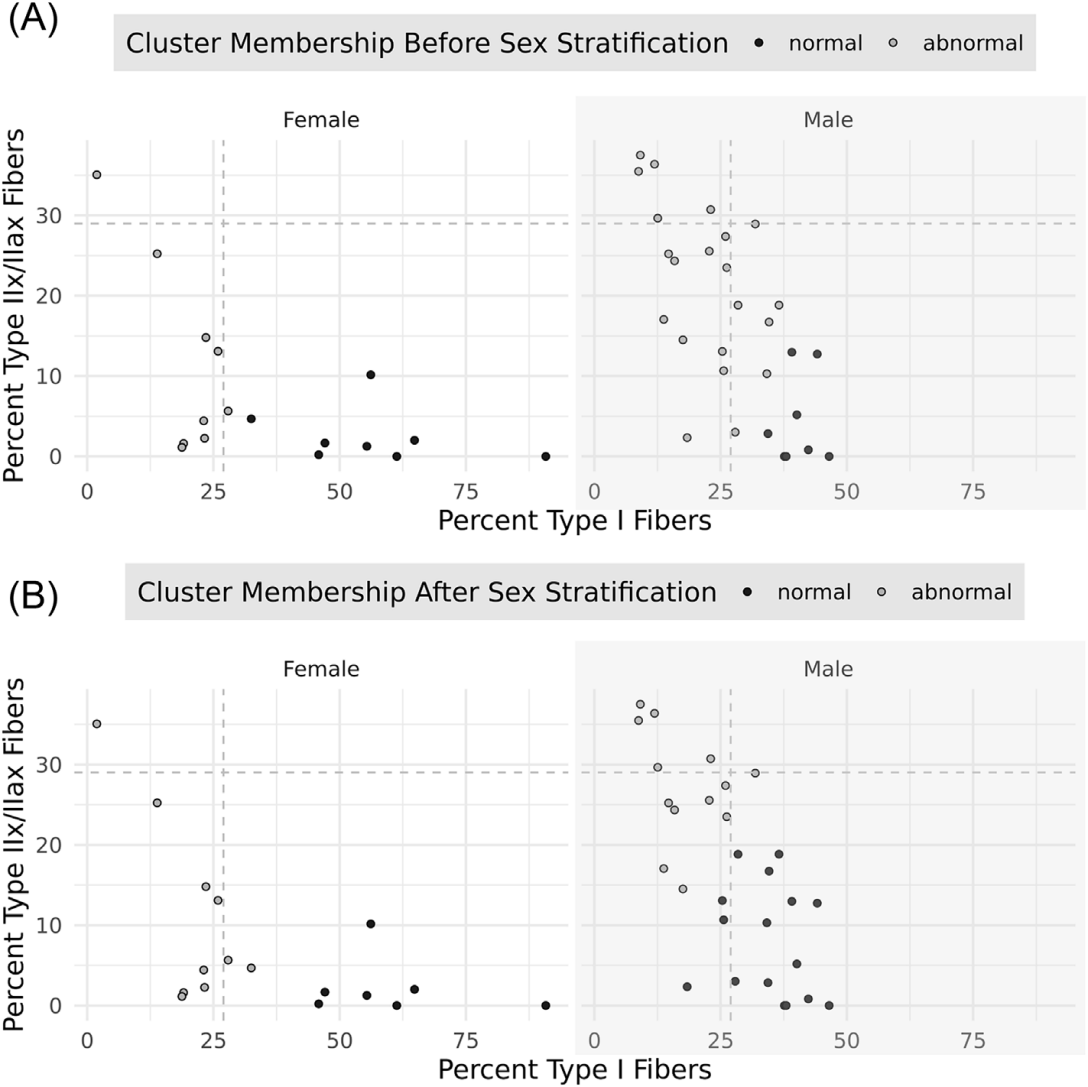
Comparison of *k*‐means clustering before and after sex stratification. Results are presented for female (left) and male (right) participants separately. The grey vertical and horizontal dashed lines represent the criteria suggested by Gosker et al.; any point above or the left of these lines represents abnormal fibre type as defined by these criteria. (A) The results of *k*‐means clustering without sex stratification: Grey dots represent patients designated as having an abnormal myofibre proportion, and black dots represent patients with a normal myofibre proportion based on this clustering system. (B) The results of *k*‐means clustering after stratifying for sex.

Unstratified clustering in participants with COPD showed no significant differences between groups in body composition, pulmonary function, or measures of strength and endurance. Using the sex‐stratified clustering approach, COPD participants with abnormal myofibre proportions had significantly lower median handgrip strength (26.1 vs. 34.0 kg, *P* = 0.022, *Table* *2*), lower median 6‐min walk distance (300 vs. 353 m, *P* = 0.039, *Table* *2*) and lower FEV_1_/FVC ratio (0.42 vs. 0.48, *P* = 0.041, *Table* *2*) than COPD participants with normal myofibre proportions. COPD participants with abnormal myofibre proportions, defined by the sex‐stratified clustering strategy, generally had <18% type I fibres and/or >22% type IIx/IIax fibres if male, or <36% type I fibres and/or more than >12% type IIx/IIax fibres if female (*Figure* *2*). Using the abnormal muscle phenotype criteria defined by Gosker et al. to classify participants, participants with COPD and abnormal myofibre proportions demonstrated greater median FMI (10.8 vs. 7.88 kg/m^2^, *P* = 0.015, *Table* *2*), greater median body mass index (BMI) (26.8 vs. 23.0 kg/m^2^, *P* = 0.020, *Table* *2*) and lower median FEV_1_ (40% vs. 49% predicted, *P* = 0.037, *Table* *2*) as compared with participants with COPD and normal fibre proportions. There were no significant associations between functional outcomes and individual myofibre proportions, whether or not sex was included as a covariate (*Table* *S3*). Of note, 35% of both male (10/28) and female (6/17) control subjects met our sex‐stratified clustering definition of abnormal myofibre proportions.

**Table 2:** Comparison of abnormal myofibre proportion definitions in participants with COPD (*N* = 46)

	Cluster normal (*n* = 16)	Cluster abnormal (*n* = 30)	*P* value	Sex‐stratified normal (*n* = 23)	Sex‐stratified abnormal (*n* = 23)	*P* value	Gosker normal (*n* = 23)	Gosker abnormal (*n* = 23)	*P* value
Demographics									
Age	60.5 (54.6–69.5)	64.8 (60.8–69.0)	0.39	64.1 (56.4–69.5)	64.8 (60.7–68.8)	0.66	62.4 (56.4–69.2)	64.8 (61.9–69.0)	0.43
Male sex	8 (50)	21 (70)	0.21	16 (70)	13 (57)	0.54	14 (61)	15 (65)	1.00
Anthropometrics									
BMI (kg/m^2^)	23.4 (20.2–26.2)	26.3 (23.1–28.1)	0.12	23.9 (21.7–27.4)	26.2 (23.2–28.5)	0.30	23.0 (21.1–26.2)	26.8 (23.9–28.8)	0.020*
SMI (kg/m^2^)	6.20 (6.11–7.96)	6.98 (6.33–7.66)	0.49	6.89 (6.16–8.01)	6.86 (5.91–7.22)	0.41	6.39 (6.12–7.78)	6.96 (6.35–7.73)	0.49
FFMI (kg/m^2^)	15.2 (13.5–18.0)	15.4 (14.0–17.0)	0.71	15.4 (14.1–18.1)	15.1 (13.4–16.1)	0.32	15.3 (13.9–17.6)	15.4 (14.2–16.9)	0.79
FMI (kg/m^2^)	7.95 (6.01–9.86)	9.39 (7.48–12.2)	0.12	8.60 (6.90–9.35)	10.8 (7.2–12.8)	0.094	7.88 (6.15–9.35)	10.8 (8.09–12.8)	0.015*
Pulmonary function									
FEV_1_, % pred.	48.4 (42.7–65.5)	45.0 (36.0–60.3)	0.37	48.0 (42.6–65.0)	43.0 (33.0–54.7)	0.14	49.0 (43.4–65.5)	40.0 (33.5–53.6)	0.037*
FEV_1_/FVC	0.48 (0.40–0.59)	0.44 (0.38–0.56)	0.35	0.48 (0.42–0.61)	0.42 (0.35–0.50)	0.041*	0.48 (0.41–0.61)	0.42 (0.37–0.50)	0.079
Functional measures									
Grip strength (kg)	26.9 (21.3–39.0)	29.5 (24.1–34.2)	0.85	34.0 (25.3–38.6)	26.1 (19.8–32.1)	0.022*	29.5 (23.6–38.6)	28.3 (19.8–34.0)	0.21
6‐min walk distance (ft)	1161 (1031–1364)	1100 (900–1200)	0.27	1159 (1090–1311)	983 (766–1211)	0.039*	1134 (977–1311)	1100 (888–1224)	0.39
30‐s sit‐to‐stand repetitions	10.0 (10.0–11.0)	11.0 (8.0–13.8)	0.63	10.0 (9.50–12.0)	10.0 (8.0–12.5)	0.83	10.0 (8.50–11.0)	11.0 (8.00–13.5)	0.49

*Note*: All data are presented as median (inter‐quartile range) unless otherwise noted.

Abbreviations: BMI, body mass index; COPD, chronic obstructive pulmonary disease; FEV_1_, % pred., fraction of exhaled volume in 1 s, per cent predicted based on subject's age, race, sex and height; FEV_1_/FVC, fraction of exhaled volume in 1 s‐to‐forced vital capacity ratio; FFMI, fat‐free mass index; FMI, fat mass index; SMI, appendicular skeletal muscle index.

* Significant differences in participants with an abnormal myofibre proportion by the various definitions.

### Differentially expressed transcripts associated with abnormal myofibre proportions in chronic obstructive pulmonary disease

**Figure 3:**
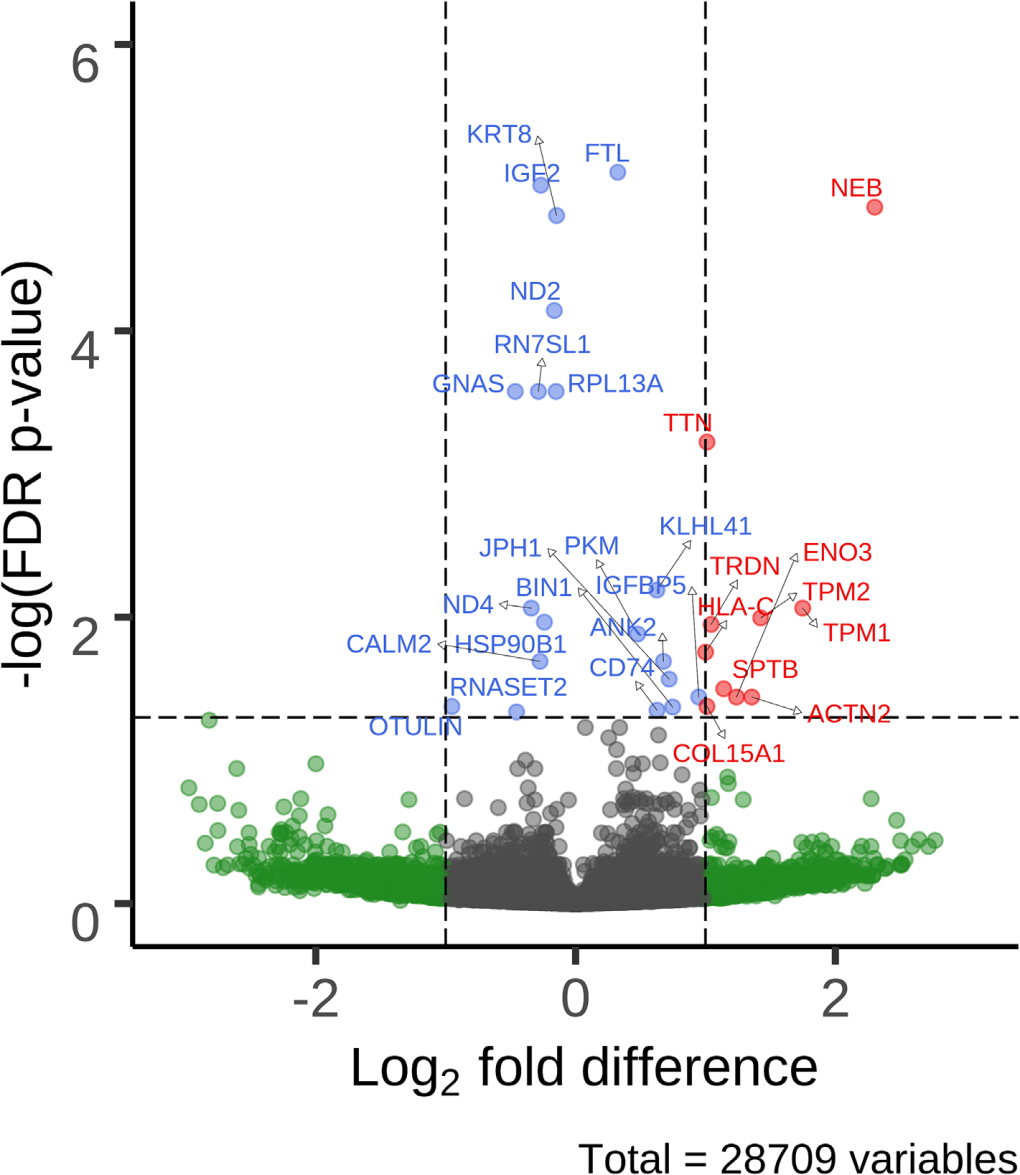
Volcano plot of differentially expressed transcripts in participants with chronic obstructive pulmonary disease associated with the presence of our sex‐stratified abnormal myofibre proportion phenotype. Labelled dots are significant after false discovery rate (FDR) correction, and labels are their respective gene names. Red dots represent transcripts with an FDR‐corrected *P* value <0.05 and an absolute log fold difference (FD) ≥1. Blue dots represent transcripts with an FDR *P* value of <0.05. Green dots represent transcripts with an absolute log FD ≥ 1.

We investigated associations between gene expression levels and our sex‐stratified abnormal myofibre proportion phenotype in participants with COPD. In total, 29 single transcripts were significantly differentially expressed between COPD participants with and without an abnormal myofibre proportion phenotype (*Figure* *3* and *Table* *3*). Transcriptomic associations with continuous myofibre proportions showed correlation with previously published myofibre‐specific gene sets,^11^, ^25^ as well as our abnormal myofibre proportion phenotype (*Tables* *S4*–*S6*). Specifically, 18 of the 29 transcripts associated with abnormal myofibre proportions were also significantly associated with at least one myofibre type as the outcome (*Table* *S7*). Similarly, most findings were robust when the Gosker et al.^2^ abnormal myofibre proportions were used; analyses were restricted to males and/or adjusted for current smoking (*Table* *S8*).

**Table 3:** Transcripts with differential expression associated with abnormal myofibre proportion

Gene	Name	Log FD	FDR *P* value	Module
*NEB*	Nebulin	2.30	1.37E − 05	Blue
*TPM1*	Tropomyosin 1	1.75	8.64E − 03	Blue
*TPM2*	Tropomyosin 2	1.43	1.01E − 02	Blue
*ACTN2*	Actinin alpha 2	1.36	3.61E − 02	Blue
*ENO3*	Enolase 3	1.24	3.61E − 02	Blue
*SPTB*	Spectrin beta, erythrocytic	1.14	3.16E − 02	Blue
*TRDN*	Triadin	1.04	1.12E − 02	Blue
*TTN*	Titin	1.01	5.98E − 04	Blue
*COL15A1*	Collagen type 15 alpha 1 chain	1.01	4.20E − 02	Blue
*HLA‐C*	Major histocompatibility complex, Class I, C	1.00	1.75E − 02	NA
*OTULIN*	OTU deubiquitinase with linear linkage specificity	−0.95	4.20E − 02	Turquoise
*IGFBP5*	Insulin‐like growth factor binding protein 5	0.95	3.61E − 02	Blue
*BIN1*	Bridging integrator 1	0.75	4.24E − 02	Blue
*JPH1*	Junctophilin 1	0.72	2.71E − 02	Blue
*ANK2*	Ankyrin 2	0.68	2.03E − 02	Blue
*CD74*	CD74 molecule	0.63	4.46E − 02	NA
*KLHL41*	Kelch‐like family member 41	0.63	6.46E − 03	Blue
*PKM*	Pyruvate kinase M1/2	0.48	1.31E − 02	NA
*GNAS*	GNAS complex locus	−0.46	2.65E − 04	NA
*RNASET2*	Ribonuclease T2	−0.45	4.58E − 02	NA
*ND4*	Mitochondrially encoded NADH:ubiquinone oxidoreductase core subunit 4	−0.34	8.64E − 03	NA
*FTL*	Ferritin light chain	0.32	7.81E − 06	NA
*RN7SL1*	RNA component of signal recognition particle 7SL1	−0.29	2.65E − 04	Grey
*CALM2*	Calmodulin 2	−0.27	2.03E − 02	NA
*IGF2*	Insulin‐like growth factor 2	−0.27	9.62E − 06	NA
*HSP90B1*	Heat shock protein 90 beta family member	−0.24	1.08E − 02	NA
*ND2*	Mitochondrially encoded NADH:ubiquinone oxidoreductase core subunit 2	−0.16	7.21E − 05	NA
*RPL13A*	Ribosomal protein L13a	−0.15	2.65E − 04	NA
*KRT8*	Keratin 8	−0.15	1.57E − 05	NA

Abbreviations: FD, fold difference; FDR, false discovery rate; NA, not included in weighted gene co‐expression network analysis due to low transcript variance.

Gene set enrichment analyses indicated that a total of 12 canonical gene sets were enriched with transcripts with differential expression associated with the presence of abnormal myofibre proportions (*Table* *S9*), including gene sets associated with myogenesis (hallmark myogenesis, FDR *P* = 1.7 × 10^−5^) and striated muscle contraction (Reactome striated muscle contraction, FDR *P* = 1.2 × 10^−7^).

### Two gene modules are positively associated with abnormal myofibre proportions

**Figure 4:**
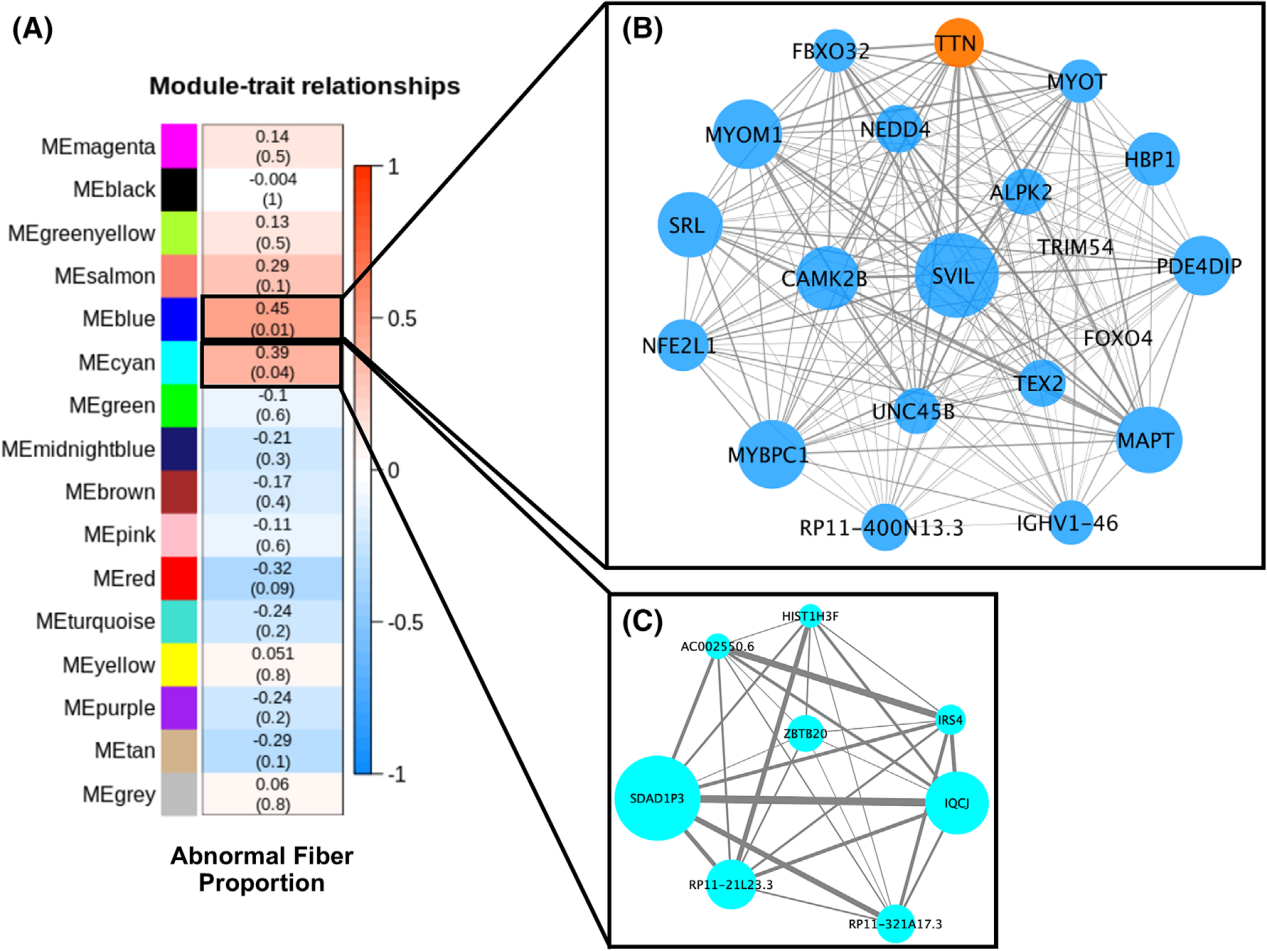
The blue and cyan modules from weighted gene co‐expression network analysis (WGCNA) are significantly positively correlated with our sex‐stratified abnormal myofibre proportion phenotype. (A) Transcriptomic module association with abnormal myofibre proportion. Values in each cell represent Pearson correlations and *P* values (in parentheses) between each module of co‐expressed transcripts and the presence of an abnormal myofibre proportion phenotype. Heatmap shading corresponds to the strength of association where darker red cells have higher upregulation and darker blue cells have higher downregulation based on correlation. Cells outlined in black are significantly associated (*P* < 0.05) with abnormal fibre type. (B, C) Network of hub transcriptomic modules significantly associated with abnormal myofibre proportion. Transcripts with a module membership (kME) >0.78 in the blue (B) and transcripts with a kME > 0.6 in the cyan (C) modules were selected for visualization, along with *TRIM54* and *FOXO4* in the blue module, due to their suspected role in skeletal muscle pathology in chronic obstructive pulmonary disease. The size of the circle in each network corresponds to increasing module membership, and the thickness of the edge corresponds to increasing topological overlap, a measure of the strength of correlation between transcript expression levels, which is Pearson's correlation obtained from the adjacency matrix. One hub transcript (TTN) in the blue module is significantly differentially expressed in the single‐transcript analysis and is coloured orange to reflect this.

Two of 16 WGCNA co‐expression modules, the blue (*r* = 0.45, *P* = 0.01) and cyan (*r* = 0.39, *P* = 0.04) modules, were significantly associated with abnormal myofibre proportion in participants with COPD (*Figure* *4* *A*). The blue module (2301 transcripts; *Figure* *4* *B* and *Table* *S10*) is significantly enriched for genes associated with muscle contraction (Reactome muscle contraction gene set, FDR *P* = 5.5 × 10^−27^), type IIa muscle fibres (gene set taken from single‐cell transcriptomics results from Rubenstein et al.,^11^ FDR *P* = 5.2 × 10^−10^), ubiquitination and proteosomal degradation (Reactome antigen processing: ubiquitination and proteasome degradation gene set, FDR *P* = 2.2 × 10^−5^), and insulin signalling (Kyoto Encyclopedia of Genes and Genomes [KEGG] insulin signalling, FDR *P* = 5.9 × 10^−5^; *Table* *S11*). Genes in the blue module were enriched with genetic variants associated with heel bone mineral density (BMD; FDR *P* = 7.5 × 10^−12^; *Table* *S12*) and pulmonary function tests (FEV_1_: FDR *P* = 2.9 × 10^−2^; FEV_1_/FVC: FDR *P* = 9.6 × 10^−3^; *Table* *S12*). Cyan module transcripts (*n* = 66; *Figure* *4* *C* and *Table* *S13*) were not significantly enriched for any known pathways and were largely comprised of transcripts not known to encode proteins.

## Discussion

Utilizing precision phenotyping that incorporates sexual dimorphism and RNA‐Seq data, this study furthers our understanding of the dynamic transcriptional environment within the skeletal muscle of participants with COPD in a mixed‐sex cohort. While skeletal muscle pathology associated with COPD may be identified by abnormal myofibre proportions, our data suggest that the involved pathophysiology is not limited to one specific fibre type within the *vastus lateralis*. We identified a large co‐expression network of transcripts that is positively associated with our abnormal myofibre proportion phenotype and is enriched with transcripts associated with both type IIa and type I fibres, despite lower type I myofibre proportion being part of the definition of this phenotype. Additionally, many transcripts that are differentially expressed on the basis of the abnormal myofibre proportion phenotype are structural in nature and highly ubiquitous. Taken together, these findings suggest that the presence of abnormal myofibre proportions is associated with a dynamic remodelling process occurring in the skeletal muscle of participants with COPD. This is consistent with prior research showing increased markers of both protein degradation and synthesis in COPD participants with and without sarcopenia, as compared with control subjects.^26^

Our differentially expressed genes conform to some elements of expected biology by aligning with prior findings of fibre‐specific differential gene expression. The transcripts *TPM1*, *ENO3* and *PKM* upregulated among those with COPD and abnormal myofibre proportions are known to be highly expressed in type II muscle fibres, consistent with how we have defined abnormal myofibre proportions.^11^ Tropomyosin 1, encoded by the *TPM1* gene, is a structural component that is upregulated in type II muscle fibres,^25^ while beta‐enolase, encoded by *ENO3*, and pyruvate kinase muscle isozyme, encoded by *PKM*, are both enzymes involved in glycolysis are upregulated in type II muscle fibres.^11^ While the prior fibre‐specific expression studies associated these transcripts with type IIa fibres, greater type II fibre‐specific transcript expression is consistent with a lower proportion of type I fibres, as seen in our abnormal myofibre proportion phenotype. Further, *TPM1*, *ENO3* and *PKM* were also significantly positively associated with type IIa fibre‐type proportion in our sensitivity analyses (*Tables* *S5* and *S7*). These results serve to confirm the face validity of our abnormal myofibre proportion phenotype.

This study also highlights the transcriptional heterogeneity in skeletal muscle tissue with mixed proportions of fibre types. Two prior studies have attempted to predict histological fibre proportion from bulk transcriptomic data. Despite clear and significant differential expression of a host of genes, these studies ultimately failed to demonstrate anything greater than moderate correlation between histological phenotype and gene expression.^11^, ^25^ One study recognized 4 of 20 samples with an intermediate expression pattern,^11^ while the other recognized two clusters of cells with indeterminate identities were proposed to represent fibroblasts and pericytes.^25^ One transcript significantly upregulated in our differential expression analysis, *COL15A1*, is also identified as strongly upregulated in this suspected fibroblast cluster.^25^ These results underscore the need to understand causes of transcriptional variation that extend beyond fibre type and possibly dispose of strict categorical assumptions about cellular identity.

The blue WGCNA module, which was positively associated with the presence of abnormal myofibre proportions in participants with COPD, underscores this transcriptional heterogeneity because it includes genes linked to both type I and type II fibres. This implies that the transcriptional environment within muscle with abnormal myofibre proportions is more complex than a simple shift away from type I fibre genes and towards type II fibre genes and instead suggests altered transcriptional regulation across fibre types. The blue WGCNA module also contains transcripts of genes previously linked to cachexia in COPD, lending support to the hypothesis that abnormal myofibre proportion may be in some ways linked to muscular atrophy.

Hub genes in WGCNA modules show high levels of connectivity to other genes within the same co‐expression module and thus have high module membership values.^23^ Because of this connectivity, hub genes can be revealing targets for further pre‐clinical research or drug repurposing. One of the transcripts with the highest module membership in the blue module is *FBXO32*, which encodes the protein atrogin‐1, an E3 ubiquitin ligase well recognized to be associated with muscle atrophy and cachexia.^27^ Another transcript with high module membership in the blue module is *NEDD4*, which encodes another E3 ubiquitin ligase that appears to be associated with post‐denervation skeletal muscle atrophy in mouse models.^28^ Long‐term cigarette smoke exposure appears to cause chronic neuromuscular junction degeneration and abnormal skeletal muscle fibre proportions in mice,^29^ suggesting that de‐innervation/re‐innervation events play a role in altering fibre proportions in the skeletal muscle of patients with COPD. The genes *FOXO1* and *FOXO4*, both included in the blue module, belong to forkhead box O family of proteins that act as mediators of muscle atrophy, with combined knockouts of *FoxO1*, *FoxO3* and *FoxO4* essentially abrogating muscle loss phenotypes in mice, although without affecting fibre distribution.^30^ Finally, *TRIM54*, the gene encoding the muscle‐specific RING‐finger family member 3 (MuRF3) protein, is also in the blue module and is associated with both fibre proportion and protein catabolism in a mouse double‐knockout model, along with a close homologue, MuRF2.^31^ Taken together, these results suggest that, within this co‐expression network, there are both signals of abnormal myofibre proportion and muscle atrophy in the muscle of participants with COPD.

The blue WGCNA module shows enrichment for genetic loci associated with heel BMD, suggesting common pathways involved in skeletal muscle and bone pathology. Recent work shows that pectoralis muscle area, an estimator of FFMI derived via computed tomography of the chest, is co‐linear with BMD measured by the same method, suggesting physiological overlap.^32^ Other studies of patients with COPD have also demonstrated this finding,^33^ suggesting a durable association that bears further mechanistic study at the molecular level.

The cyan WGCNA module is more challenging to interpret, as it includes large numbers of transcripts that do not encode proteins. Some of these untranslated RNAs are long, non‐coding RNAs, which likely have some regulatory effects in muscle that are not currently fully understood.^34^ There are also micro‐RNAs present in the cyan module, a class of untranslated RNAs that have more well‐defined effects on skeletal muscle development and regeneration.^35^ Though the cyan module does not contain any of the canonical skeletal muscle‐specific micro‐RNAs, it does include miR‐208a, which is recognized as a regulator of cardiac hypertrophy.^36^ Further research may illuminate the importance of the non‐coding RNAs within the cyan module.

By demonstrating the utility of sex‐stratified clustering to define a pathological phenotype within a broader chronic disease population, this study underscores the importance of considering sexual dimorphism in muscle research. While our cut‐offs for diagnosing abnormal myofibre proportions in male participants are similar to the historical cut‐offs proposed by Gosker et al.,^2^ the cut‐offs for female participants are significantly different. Our sex‐stratified model showed associations with differences in lung function and functional status (6‐min walk distance, handgrip strength and FEV_1_/FVC ratio), as opposed to body composition and lung function (BMI, FMI and FEV_1_) in the criteria proposed by Gosker et al.^2^ This demonstrates that accounting for sex effects can capture differences in clinically relevant domains. The use of *k*‐means clustering, as employed in this study, can help to dichotomize outcomes across the spectrum from normal to abnormal myofibre proportions, but it is important to note that the cut‐offs we used may not be externally generalizable depending on the characteristics of the population being studied.

It is interesting that the historical criteria demonstrate an association with increased FMI and BMI, which is mirrored in direction but not significance by our abnormal myofibre proportion phenotype. The general pattern of reduced type I and increased type IIx/IIax fibres is consistent with patterns induced by forced inactivity in healthy participants, which can be exacerbated by hypoxaemia.^37^ When combined with the observation that participants with abnormal myofibre proportions have reduced functional status, this may suggest that these abnormal myofibre proportions are, at least in part, a cellular representation of reduced physical activity due to disease severity, which subsequently begets reduced physical activity in a cyclical pattern. This observation may also suggest a remedy: increasing physical activity, such as participation in a pulmonary rehabilitation programme. Prior research has shown a reduction in type IIx/IIax fibres and an increase in type I fibres after pulmonary rehabilitation in patients with COPD, which was consistently observed across the disease severity spectrum.^3^ These findings also revealed improvements in functional capacity, as measured by 6‐min walk distance and peak work rate on exercise testing, supporting the established link between myofibre composition and exercise tolerance.^3^ This finding is in keeping with our observation of significantly lower function in participants with abnormal myofibre proportions and suggests that this phenotype is potentially reversible. Further research is needed to identify potential countermeasures to abnormal myofibre proportions in COPD.

This study has significant strengths: chiefly, the rigorous phenotyping of participants with COPD across multiple dimensions, including body composition, functional status, muscle histology and muscle transcriptomics. We included a greater percentage of female participants and more participants with less severe COPD than previous studies in our histological analyses, which may explain some of the attenuated differences between control and COPD participants seen in our study. We implemented unsupervised and agnostic methods where possible. Our findings are generally consistent with prior studies with the one exception that we have demonstrated the importance of considering sex, which is a major strength of this study. We also demonstrated that transcripts associated with abnormal myofibre proportion results were robust across sensitivity analyses, including restricting the analyses to male participants.

Our study also has limitations. Although we accounted for sexual dimorphism in our definition of abnormal myofibre proportions, our transcriptomic analyses were limited to a group of 29 participants with COPD, of whom only eight were women. While the results of a sensitivity analysis in male participants were consistent with the overall results, we cannot rule out the influence of sex imbalance on our transcriptomic results. Further, we examined transcriptional changes in bulk skeletal muscle samples rather than fibre type‐specific or single nuclei level RNA‐Seq, leaving opportunities to refine our understanding of the cellular heterogeneity of skeletal muscle in participants with COPD. Despite our inclusion of a greater percentage of participants with less severe COPD, we were unable to rigorously compare this physiology across GOLD stages of severity due to insufficient cohort size, leaving an opportunity for further study when more transcriptomic data are available. Our histological findings do not fully reproduce prior research demonstrating clear evidence of differences in type I myofibre proportions between participants with and without COPD,^2^, ^8^ which could be due to a number of factors, including the inclusion of less severe COPD participants, a smaller sample size and the use of a research population from the Southern United States, which may have lower average levels of physical activity compared with the European populations previously studied.^38^ Although we performed sensitivity analyses, it was not possible to disentangle the role of current smoking on the transcriptomics results (*Table* *S8*). While current smoking is known to affect the transcriptome,^39^ these effects are confounded by a negative correlation between smoking and comorbidity, where patients with higher levels of comorbidity are often less likely to continue to smoke.^40^ Finally, this study uses invasive biopsy measures to assess for skeletal muscle phenotypes, but less invasive methods, such as imaging or blood sampling, would be much preferable to patients. The development of less invasive test methods for skeletal muscle phenotype in COPD would significantly advance detection in the clinical setting.

This study demonstrates that COPD muscle fibre proportion criteria that incorporate sexual dimorphism in fibre predominance can improve the discrimination of patients on the basis of functional outcomes. Our transcriptomics analyses are consistent with prior knowledge of myofibre‐specific gene expression but also serve to underscore the residual transcriptional heterogeneity present within skeletal muscle tissue, demonstrating the need for spatial and single‐nucleus transcriptomic studies in patients with COPD. Further research is needed to better characterize how sex differences, COPD severity and progression, and the presence of comorbidities might affect fibre‐type proportions and/or the skeletal muscle micro‐environment. Finally, further longitudinal study is needed to determine how the abnormal myofibre proportion phenotype is related to other important skeletal muscle comorbidities in COPD, such as cachexia, sarcopenia and wasting.

## Conflict of interest statement

HBR reports consulting fees from the NIH RECOVER‐ENERGIZE Working Group (1OT2HL156812) and is involved in contracted clinical research with United Therapeutics, Genentech, Regeneron, Respira and Intervene Immune. He is a visiting professor at the University of Leeds, UK. The remaining authors declare that they have no conflicts of interest.

## Supporting information


**Table S1.** Comparison of the Histology Cohort and the Subcohort with both Histology and RNA sequencing Data. All data presented as mean (standard deviation) unless otherwise noted. 6‐min. walk dist. – Six‐minute walk distance. BMI – body mass index. FEV1% predicted – fraction of exhaled volume in one second, percent predicted based on subject's age, race, sex, and height. FEV1/FVC – ratio of fraction of exhaled volume in one second to forced vital capacity. FFMI – fat free mass index. FMI – fat mass index. Knee Ext. Strength – knee extension strength. SMI – skeletal muscle index.
**Table S2.** Histology Data by Sex and COPD Disease Status. All data presented as mean (standard deviation) unless otherwise noted. * ‐ denotes significant difference with p value < 0.05. CSA ‐ cross‐sectional area, MFA ‐ mean fiber area.
**Table S3.** Linear Models of Functional Outcomes and Fat Mass Index based on Fiber Proportions. All models were constructed as linear models of functional outcome based on each specific fiber type, with or without sex as a covariate. Models with significant p‐values are denoted by an asterisk (*).
**Table S4.** Differential Gene Expression Analysis of Type I Fiber Proportion. In addition to the analysis results, we have provided comparison with canonical Type I fiber and Type IIa fiber gene sets from Rubenstein et al (2020) and Oskolkov et al. (2022) as well as the results of our differential expression analysis of the abnormal muscle phenotype trait. FD ‐ fold difference; FDR p‐value ‐ false‐discovery rate adjusted p‐value.
**Table S5.** Differential Gene Expression Analysis of Type IIa Fiber Proportion. In addition to the analysis results, we provide comparisons with canonical Type I fiber and Type IIa fiber gene sets from Rubenstein et al (2020) and Oskolkov et al. (2022) as well as the results of our differential expression analysis of the abnormal muscle phenotype trait. FD ‐ fold difference; FDR p‐value ‐ false‐discovery rate adjusted p‐value.
**Table S6.** Differential Gene Expression Analysis of Type IIx/IIax Fiber Proportion. In addition to the analysis results, we have provided comparison with canonical Type I fiber and Type IIa fiber gene sets from Rubenstein et al (2020) and Oskolkov et al. (2022) as well as the results of our differential expression analysis of the abnormal muscle phenotype trait. FD ‐ fold difference; FDR p‐value ‐ false‐discovery rate adjusted p‐value.
**Table S7.** Differentially Expressed Genes and Transcriptomic Associations with Individual Myofiber Proportions. Comparison between differentially expressed genes on the basis of our abnormal myofiber proportion phenotype collated to significant results on the basis of individual myofiber proportions. FD ‐ fold difference; FDR p‐value ‐ false‐discovery rate adjusted p‐value.
**Table S8.** Differential Gene Expression Analysis of Other Clustering Models. In addition to the analysis results, we provide comparisons to our differential expression analysis of the abnormal muscle phenotype trait. FD ‐ fold difference; FDR p‐value ‐ false‐discovery rate adjusted p‐value.
**Table S9.** Canonical Pathway (*n* = 11), Fiber Type (n = 1) and Transcription Factor Target (*n* = 51) Gene Sets Enriched for Significantly Differentially Expressed Genes (DEGs). N Genes ‐ number of genes in the pathway, N Overlap ‐ number of genes in this pathway that overlap with the blue module transcripts, FDR p‐value ‐ false‐discovery rate corrected p‐value for the overlap.
**Table S10.** Blue WGCNA Module genes (*n* = 2,301) with associated p‐values from the differential expression analysis, sorted from highest module membership to lowest. MEblue ‐ module membership in the blue module. logFC ‐ log 2 of the fold change between participants with and without frame shift. adj. p. val. ‐ Benjamini‐Hochberg corrected p value.
**Table S11.** Canonical Pathway (*n* = 61) and Fiber Type Gene Sets (*n* = 2) Enriched for Blue WGCNA Module Genes. N Genes ‐ number of genes in the pathway, N Overlap ‐ number of genes in this pathway that overlap with the blue module transcripts, FDR p‐value ‐ false‐discovery rate corrected p‐value for the overlap.
**Table S12.** GWAS Loci Identified in Other Conditions (*n* = 45) Enriched for Blue WGCNA Module Genes. GWAS ‐ genome wide association study, N Genes ‐ number of genes with loci identified by GWAS, N Overlap ‐ number of genes in these GWAS results that overlap with the blue module transcripts, FDR p‐value ‐ false‐discovery rate corrected p‐value for the overlap.
**Table S13.** Cyan WGCNA Module genes (*n* = 66) with associated p‐values from the differential expression analysis, sorted from highest module membership to lowest. MEcyan ‐ module membership in the cyan module. logFC ‐ log 2 of the fold change between participants with and without frame shift. adj. p. val. ‐ Benjamini‐Hochberg corrected p‐value.

**Figure S1.** MA plot of the transcriptional analysis of the abnormal myofiber proportion phenotype. Dots represent each individual transcript, with mean expression on the x axis and fold change on the y axis. Colored dots have a false discovery rate p‐value < 0.05 and a log fold change > 0.1. Red dots represent transcripts with positive fold changes, while blue dots represent transcripts with negative fold changes. Each transcript with a significant p‐value is labeled with its gene symbol.
