# Supplementary figures and images for: Differentially co‐expressed myofibre transcripts associated with abnormal myofibre proportion in chronic obstructive pulmonary disease
Source: J Cachexia Sarcopenia Muscle. 2024 Apr 22;15(3):1016–29. doi: 10.1002/jcsm.13473 (PMC11154789; doi:10.1002/jcsm.13473)

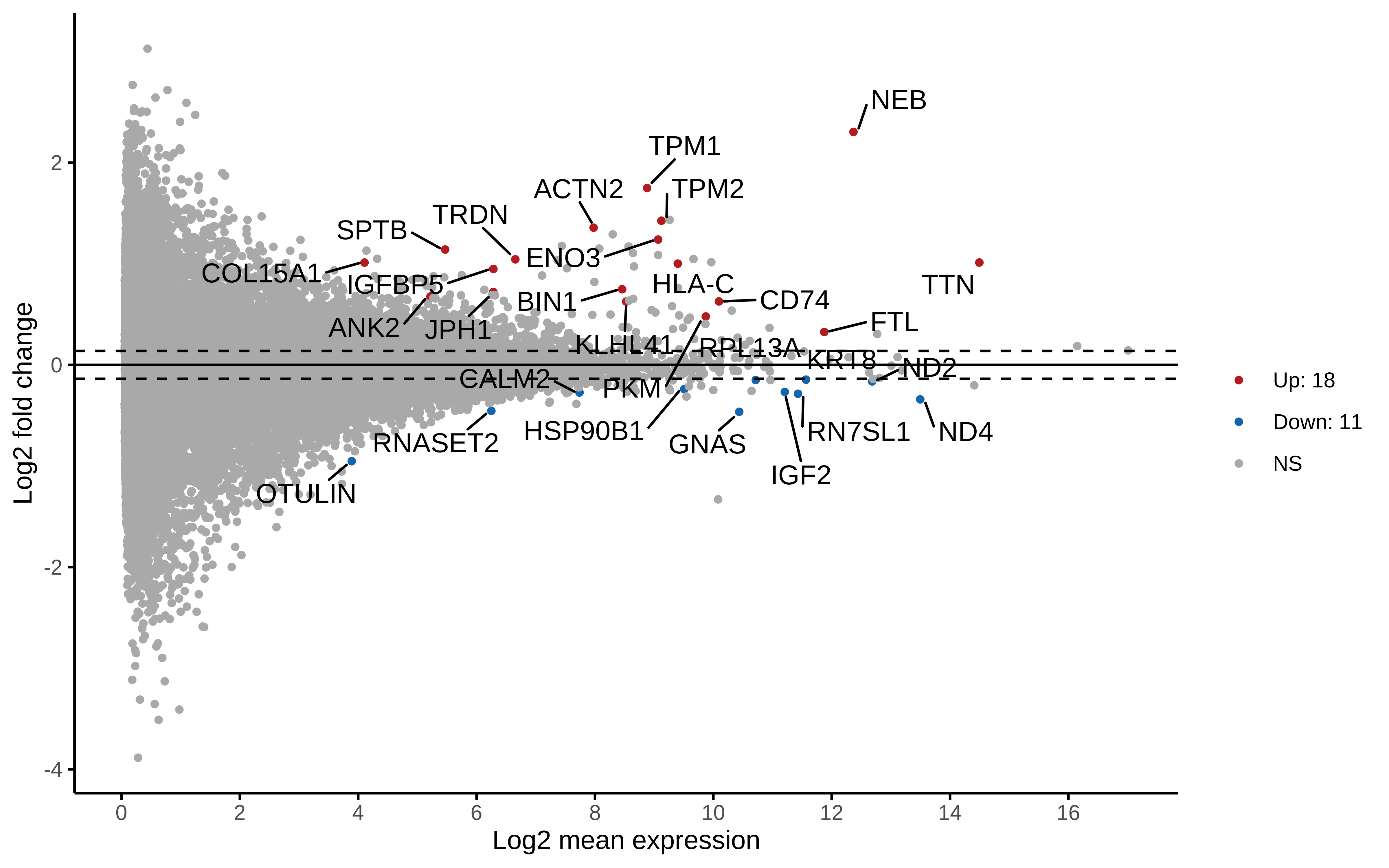

Supplement: Supplementary file 2 — Figure S1. MA plot of the transcriptional analysis of the abnormal myofiber proportion phenotype. Dots represent each individual transcript, with mean expression on the x axis and fold change on the y axis. Colored dots have a false discovery rate p‐value < 0.05 and a log fold change > 0.1. Red dots represent transcripts with positive fold changes, while blue dots represent transcripts with negative fold changes. Each transcript with a significant p‐value is labeled with its gene symbol. [file JCSM-15-1016-s001.tif]
